# Thickness-dependent optimization of Er^3+ ^light emission from silicon-rich silicon oxide thin films

**DOI:** 10.1186/1556-276X-6-395

**Published:** 2011-05-25

**Authors:** Sébastien Cueff, Christophe Labbé, Olivier Jambois, Blas Garrido, Xavier Portier, Richard Rizk

**Affiliations:** 1 Centre de Recherche sur les Ions, les Matériaux et la Photonique (CIMAP), ENSICAEN, CNRS, CEA/IRAMIS, Université de Caen, 14050 CAEN cedex, France; 2 Departament Electrònica, MIND-IN2UB, Universitat de Barcelona, Martí i Fanquès 1, 08028 Barcelona, CAT, Spain

## Abstract

This study investigates the influence of the film thickness on the silicon-excess-mediated sensitization of Erbium ions in Si-rich silica. The Er^3+ ^photoluminescence at 1.5 μm, normalized to the film thickness, was found five times larger for films 1 μm-thick than that from 50-nm-thick films intended for electrically driven devices. The origin of this difference is shared by changes in the local density of optical states and depth-dependent interferences, and by limited formation of Si-based sensitizers in "thin" films, probably because of the prevailing high stress. More Si excess has significantly increased the emission from "thin" films, up to ten times. This paves the way to the realization of highly efficient electrically excited devices.

## Background

The realization of efficient Si-based optical emitters for photonics is one of the most challenging objectives for the semiconductor community [1]. Such a purpose is confronted to the indirect band gap of bulk silicon which makes difficult the light emission from Si, and then presents a major obstacle to full photonic-electronic integration. However, the indirect sensitization of emission from erbium ions, via Si nanoclusters (Si-nc), in the technologically important 1.5-μm spectral region is a promising approach that has received significant attention. Such a sensitizing effect of Si-ncs increases the effective excitation cross section of Er by 10^3^-10^4 ^over a broad band in Si-rich silicon oxide (SRSO) systems [2]. This leads to the observation of enhanced Er photoluminescence (PL) and electroluminescence in the standard telecommunications wavelength band around 1.54 μm [2,3]. Depending on the targeted application, the thickness of the active layer can vary over a large range, from a micrometer-scale for planar waveguide amplifiers [4] to a few tens of nanometers for electrically driven LEDs [3] or slot waveguides [5]. According to recent studies, layer thickness was shown to influence the nucleation and growth of Si-ncs [6-8], as well as the effective intensity of the pump beam [9] and the local density of optical states (LDOS) [10,11]. This thickness dependence is crucial since each application requiring a given thickness may necessitate a specific optimization of the material.

In this paper, we investigate the impact of layer thickness on the optical properties of SRSO:Er thin films. The results demonstrate that the photoluminescence in very thin layers is hindered by some thinness-related limiting factors. To overcome this drawback of thin layer, more Si excess was gradually incorporated until a level of Er emission that was found surprisingly higher than that observed in optimized micrometer-thick layers.

### Experimental details

The SRSO films doped with Er were grown onto a p-type, 250-μm thick, (100) silicon wafer, by magnetron co-sputtering of three confocal cathodes (SiO_2_, Si and Er_2_O_3_) under a plasma of pure Argon at a pressure of 2 mTorr. The power densities applied on the three confocal targets were kept constant, while the deposition was performed at two temperatures *T*_d_, room temperature (RT) and 500°C, for various durations between 20 min and 10 h. To examine the influence of Si excess for a set of thin films of about 50 nm in thickness, the power density on the Si target was subsequently increased. The thickness and refractive index *n *were measured by spectroscopic ellipsometry for films thinner than 500 nm and by m-lines techniques for films exceeding 500 nm in thickness. The thickness shows a linear variation with the deposition duration. The PL spectra were recorded using the non-resonant 476-nm excitation wavelength in order to ensure that Er^3+ ^ions are only excited through the sensitizers. The samples were excited with 45° incident spot of approximately 3 mm^2 ^with a power of 180 mW, *i.e.*, a power density of 0.06 W/mm^2^. The Er content was obtained by time-of-flight secondary ion mass spectroscopy technique after calibration by a reference SRSO:Er sample containing a known Er concentration. The erbium concentration was found nearly constant for all samples at about 3 × 10^20 ^at. cm^-3^. The Si excess was evaluated by two methods: X-ray photoelectron spectroscopy (XPS) exploring beyond 100-nm depth (or total thickness for thinner films) in different places, and Fourier transform infrared (FTIR) spectroscopy with a spot covering a large area of the sample. Transmission electron microscopy (TEM) observations were performed using a JEOL 2010F operated at 200 kV.

## Results

**Figure 1:**
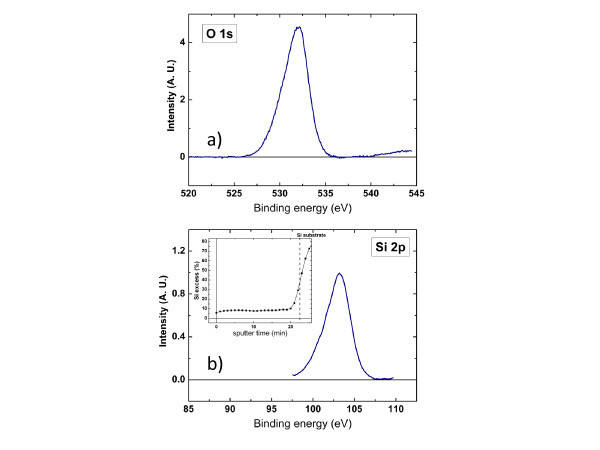
**Typical XPS spectra obtained on the sample deposited at 500°C and about 150 nm thick**. In (**a**) is displayed the O 1*s *spectrum and (**b**) corresponds to Si 2*p *spectrum. The inset of (b) depicts the profile of %Si excess *versus *depth.

**Figure 2:**
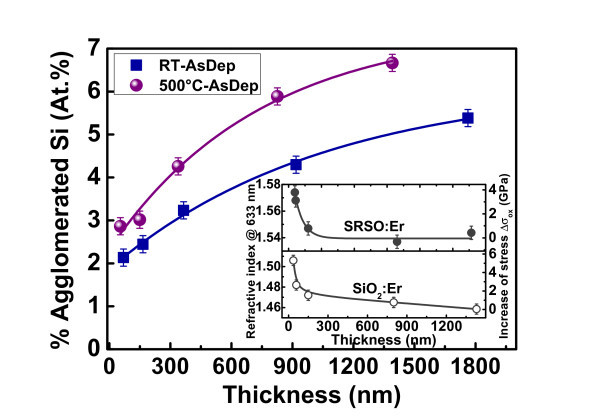
**Evolution of the estimated atomic percentage of agglomerated Si as a function of the film thickness**. For as-deposited SRSO:Er layers deposited both at room temperature and at 500°C. The lines are guides to the eye. Inset: evolution of the refractive index and estimated increase of the compressive stress (right scale) for SiO_2_:Er and SRSO:Er as a function of the thickness.

Typical Si 2*p *and O 1*s *XPS spectra of the sample deposited at 500°C for 1 h are displayed in Figure 1. The values of Si excess were determined by measurement of the ratios of the atomic concentration of Si and O (*x *= [O]/[Si]), that were deduced from the area of the Si 2*p *and O 1*s *spectra and compared to a stoichiometric SiO_2 _sample. The XPS measurements are performed while etching the sample with Ar in the same time, allowing the determination of the Si excess depth profile. The reported values correspond to the value read in the flat region (see inset Figure 1b). For the thinner layer, the thickness is still large enough to be able to obtain a good depth resolution. The flatness of the profiles along almost the whole thickness demonstrates that the thickness of the material has no influence on the stoichiometry of the deposited SiO_*x*_. However, the *x *parameter was found to increase from *x *= 1.555 ± 0.004 for RT-deposited samples to *x *= 1.616 ± 0.009 for *T*_d _= 500°C. This reflects a lowering of Si excess due to the increasing desorption of SiO with *T*_d_, as observed in our recent work [12]. For the FTIR approach, which is based on the shift of the TO_3 _peak towards that of stoichiometric SiO_2 _[13], the detection of Si excess is limited to the Si atoms bonded to O, and does not take into account the agglomerated Si atoms [13]. However, this limitation can be used to advantage by comparing values of Si excess measured by FTIR to those determined by XPS, enabling evaluation of the fraction of agglomerated Si. Since the phase separation between Si and SiO_2 _is incomplete for the as-deposited samples, the following relation holds:(1)

with *y *the stoichiometry parameter (SiO_*y*_) detected by FTIR, implying *x *<*y *< 2. The atomic percentage of agglomerated Si, %Si_agglo_, can be estimated from *((y *- *x)/y)/(1 + x) *and its evolution with thickness is shown in Figure 2 for the two series deposited at RT and 500°C. A single isolated Si atom is highly likely not able to act as a sensitizer, therefore this parameter (%Si_agglo_) includes the total population of Si-based sensitizers consisting in either Si-ncs, the so-called luminescent centers of Savchyn *et al. *[14], or the atomic scaled agglomerates suggested recently by our group [15]. To effectively play their sensitizing role, these entities should be located at less than about 1 nm of an optically active Er ion. Figure 2 shows that the agglomeration of Si is favored by increased *T*_d _and/or film thickness. While the raise of *T*_d _from RT to 500°C is expected to enhance the clustering of silicon during deposition, the most striking aspect is the pronounced increase of %Si_agglo _*versus *thickness. Note that the fraction of agglomerated Si in both RT-deposited and 500°C-deposited samples shows a similar increasing trend, but less pronounced for the former one, suggesting that this phenomenon stems from the influence of the thickness. Such an influence has been demonstrated earlier and assigned to the existence of a nucleation barrier for the formation of Si-nc as a function of the separation distance from the substrate, *i.e. *the film thickness [6-8]. This barrier is likely induced by the stress that is inversely proportional to film thickness [16], and thus prevents a complete phase separation of the SiO_*x *_system [17]. For an unchanged stoichiometry, the relative evolution of the internal stress of SiO_2 _deposited on Si substrate has been linked to its refractive index by the following relation [18]:(2)

with *n*(σ_ox_) the refractive index for a given thickness, *n*_0 _the refractive index for relaxed or "bulk" SiO_2 _(1.458) and Δ*n*/Δσ_ox _= 9.10^-12 ^Pa^-1^, taken from Ref. [18]. The inset in Figure 2, shows a pronounced increase of *n *for a range of our thin films (<150 nm) for both matrix (SiO_2 _and SRSO) and is similar to that reported in Ref. [18], hence attesting of a thickness-dependent stress. The stress difference can be estimated to 4-6 GPa between the thinnest and thickest films. The main origin of this internal stress arises from the misfit between the substrate and the film. Its progressive increase when the films' thickness is reduced seems to inhibit the agglomeration of Si.

**Figure 3:**
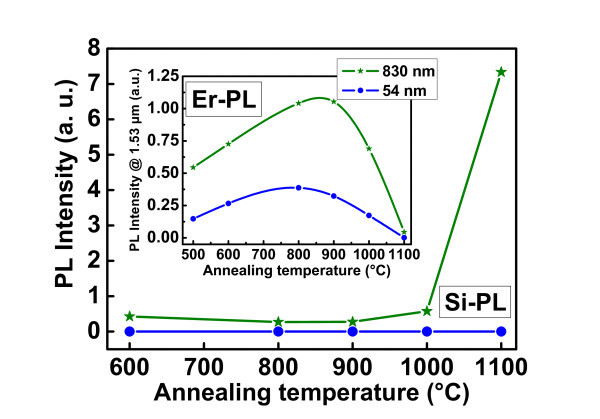
**Evolution of the integrated PL visible emission as a function of the annealing temperature**. For two typical thicknesses (54 and 830 nm) of the samples deposited at 500°C. The inset displays the evolution of the corresponding Er PL intensity at 1.54 μm (normalized to film thickness) as a function of annealing temperature.

**Figure 4:**
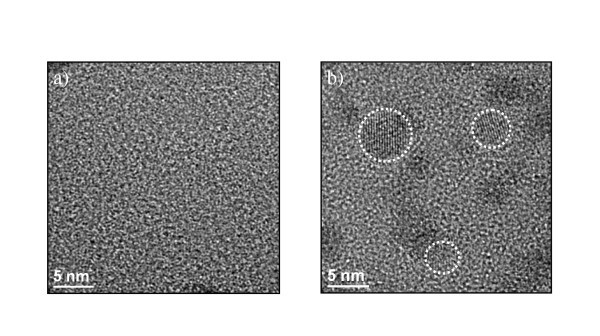
**Transmission electron microscope images, of samples deposited at 500°C for two different thicknesses**. (**a**) 50 nm and (**b**) 1,400 nm. In "thin" film (a) no Si-nc was detected throughout the whole area of the sample, while in "thick" film (b) numerous well-crystallized Si-ncs are seen with diameter as high as 5 nm. The observed darker regions in (b) are accounted for Er-clusters and are observed also in some regions of "thin" films.

**Figure 5:**
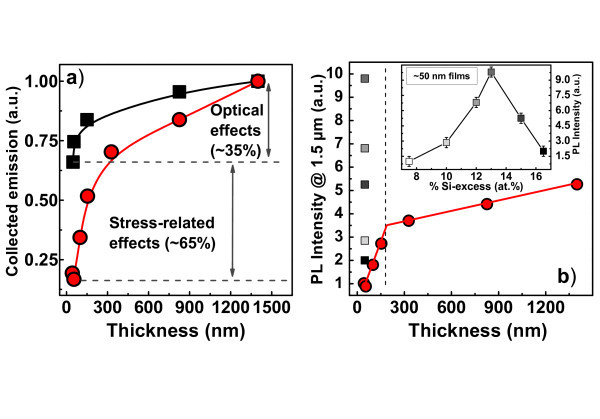
**The calculated intensity *I***_**cal **_**is compared to *I***_**PL **_**and evolutions of *I***_***PL***_. (**a**) Evolution of the experimental Er PL Intensity at 1.54 μm, IPL (circles), and calculated *I*_cal _(squares) due to LDOS and interference effects (see text), as a function of film thickness. For the sake of comparison, both intensities are normalized to the highest values at 1,400 nm. (**b**) Variation of *I*_PL _for 7.5 at.% of Si excess (circles) and for 50-nm-thick films with a varying Si excess (gray-scale squares). Inset: *I*_PL _in function of Si excess for thin samples of about 50 nm.

Accordingly, the PL properties of typical "thin" and "thick" layers deposited at 500°C can be compared. Figure 3 shows typical variations of the PL intensity (normalized to the thickness) of emission, both from Si-ncs around 750 nm, and from Er ions around 1.5 μm (see inset), as a function of the annealing temperature (*T*_a_). The influence of *T*_a _on the agglomeration of Si excess was previously studied [19] and it was shown that the value of %Si_agglo _increases almost linearly *versus T*_a _before reaching a complete agglomeration at 1,100°C, whatever the temperature of deposition and the %Si_excess_. Three major observations can be made: (1) Er PL shows the same evolution for both "thin" and "thick" samples, with an optimum for *T*_a _= 900°C, (2) The Si-nc-PL detected from the thick sample rises spectacularly for *T*_a _= 1,100°C. This opposite behavior of the Si-nc and Er emissions for thick films has been already observed and explained [20,21]. By contrast, no Si-nc PL emission is detected from the thin films, even after a 1,100°C annealing. This phenomenon is due to the low fraction of agglomerated Si (see Figure 2), and is confirmed in Figure 4 by TEM images of both thin and thick samples annealed at 1,100°C that shows the presence of well-defined crystallized Si-ncs in thick samples but not in the thin one. Such inhibition of the nucleation of Si-nc in thin films was already assumed in several studies based on PL results [6,10] but these TEM images are direct evidence of this phenomenon. (3) The Er emission is almost four times lower for the thin sample for all *T*_a_. Such a gap between the Er PL from the "thin" and "thick" samples deserves further attention. The above-mentioned limitations (stress) and depth-dependent optical effects (LDOS, interference) related to the film thinness are to be circumvented and/or considered. To estimate the impact of both interference-induced variations of the pumping and LDOS effects, we made calculations based on the methods described in Refs. [9] and [10], respectively. Their specific contributions at a distance z from the substrate were then estimated, and their product integrated over the thickness has allowed the calculation of their combined contributions, *I*_cal_, on the measured Er PL intensity, *I*_PL_. The calculated intensity *I*_cal _is compared in Figure 5a to *I*_PL_. For the sake of comparison, both *I*_cal _and *I*_PL _are normalized to the highest values, at 1,400 nm where the stress effect on the Er PL intensity can be relatively neglected. While *I*_PL _shows an abrupt decrease at about 200 nm, indicated by the vertical dashed line of Figure 5b, *I*_cal _shows a smaller reduction down to a level significantly higher than the corresponding level for *I*_PL_. An approximately five-time lowering of *I*_*PL *_and nearly 1.5 times decrease of *I*_*cal *_occur at the thickness threshold of approximately 200 nm, beyond which the above-mentioned limitations are less effective. The additional reduction of *I*_PL_, compared to *I*_cal _can be attributed to a stress effect which affects the formation and homogeneity of the sensitizers.

To overcome these limitations, we have gradually raised the Si excess in approximately 50-nm-thick films, with the objective of increasing the number of Si-based sensitizers. We show in Figure 5b the evolutions of *I*_*PL *_containing approximately 7.5 at.% Si excess (circles) as a function of the film thickness and *I*_*PL *_of thin films (approximately 50 nm) with different Si excess (squares) for the samples processed using optimized conditions (*T*_d _= 500°C, *T*_a _= 900°C, see Figure 3).

We plot in the inset of Figure 5b the evolutions of *I*_*PL *_in function of the Si excess for the 50-nm-thick films. The *I*_*PL *_optimum is reached for about 14 at.%, before decreasing for higher Si contents. In parallel, we observe a gradual and systematic decrease of the lifetime of Er emission, from nearly 1.8 ms to about 1 ms (not shown). This reflects the creation of new non-radiative decay channels [22], which should attenuate the Er PL. For Si excess lower than 14 at.%, such an attenuation is somehow dominated by the increase of excitation of Er^3+ ^ions through more sensitizers. Beyond 14 at.%, the new non-radiative decay channels start to dominate, leading to the observed decline of Er PL [22]. The Er PL peak intensity is ten times that of the similar thin film containing 7.5 at.% excess Si, and five times that observed for optimized thick samples containing 7.5 at.% excess Si (see corresponding symbols at the left part of Figure 5). Such an optimisation of the Si excess for 1-μm-thick samples was made earlier [15]. The optimum Si excess in these 50-nm-thick films is almost twice the excess incorporated in the best thin layers studied so far by our team [3]. This offers the double advantage of minimizing the limiting factors present in thin films, and favoring the transport of electrically injected carriers. In addition, the proportion of Er ions coupled to sensitizers is likely to be significantly improved, allowing one to expect a fraction of inverted Er much higher than the reported 20% [3].

## Conclusions

In summary, the influence of layer thickness on the photoluminescence of Er ions has been investigated for SRSO:Er layers. It was shown that thinness-related effects decrease the PL for thin films by a factor of 5. These effects are mainly due to three origins: (1) high stress prevailing in thin films that inhibits the formation of Si nanoclusters, (2) changes in LDOS, and (3) changes in the pumping rates. To minimize the thinness-related limitations in thin films, the amount of Si excess was gradually increased until reaching an Er PL intensity one order of magnitude higher than that recorded earlier for similar thin samples. Such a route appears very promising for the improvement of electrically driven high-performance Si-based light sources.

## Competing interests

The authors declare that they have no competing interests.

## Authors' contributions

SC fabricated the samples and performed the experiments, except SIMS and XPS measurements made by OJ who also helped in the estimate of agglomerated Si. CL made the calculations dealing with the effects of interferences and local density of optical states, in addition to specific contributions in each steps of the study. XP carried out the TEM experiments. BG participated to the finalization of the manuscript. RR drafted the manuscript, together with contributions to the analysis of the results. All authors discussed and commented on the manuscript.

## References

[B1] Daldosso N Pavesi L Nanosilicon photonics Laser & Photonics Reviews 2010 3 508 53421618721

[B2] Kenyon AJ Trwoga PF Federighi M Pitt CW Optical properties of PECVD erbium-doped silicon-rich silica: evidence for energy transfer between silicon microclusters and erbium ions J Phys: Condens Matter 1994 6 L319 10.1088/0953-8984/6/21/007

[B3] Jambois O Gourbilleau F Kenyon AJ Montserrat J Rizk R Garrido B Towards population inversion of electrically pumped Er ions sensitized by Si nanoclusters Opt Exp 2010 18 2230 10.1364/OE.18.002230 20174051

[B4] Daldosso N Navarro-Urrios D Melchiorri M García C Pellegrino P Garrido B Sada C Battaglin G Gourbilleau F Rizk R Pavesi L Er-coupled Si nanocluster waveguide IEEE J Sel Top Quant Electron 2006 12 1607

[B5] Barrios CA Lipson M Electrically driven silicon resonant light emitting device based on slot-waveguide Opt Exp 2005 13 10092 10.1364/OPEX.13.010092 19503222

[B6] Fang YC Li WQ Qi LJ Li LY Zhao YY Zhang ZJ Lu M Photoluminescence from SiO_*x *_thin films: effects of film thickness and annealing temperature Nanotechnology 2004 15 494 10.1088/0957-4484/15/5/016

[B7] Ahmad I Temple MP Kallis A Wojdak M Oton CJ Barbier D Saleh H Kenyon AJ Loh WH Silicon nanocluster-sensitized emission from erbium: The role of stress in the formation of silicon nanoclusters J Appl Phys 2008 104 123108 10.1063/1.3050324

[B8] Zacharias M Streitenberger P Crystallization of amorphous superlattices in the limit of ultrathin films with oxide interfaces Phys Rev B 2000 62 8391 10.1103/PhysRevB.62.8391

[B9] Ferre R Garrido B Pellegrino P Peràlvarez M Garcia C Moreno JA Carreras J Morante JR Optical-geometrical effects on the photoluminescence spectra of Si nanocrystals embedded in SiO_2_ J Appl Phys 2005 98 084319 10.1063/1.2115100

[B10] Kalkman J Gersen H Kuipers L Polman A Excitation of surface plasmons at a SiO_2_/Ag interface by silicon quantum dots: Experiment and theory Phys Rev B 2006 73 075317

[B11] Horak P Loh WH Kenyon AJ Modification of the Er^3+ ^radiative lifetime from proximity to silicon nanoclusters in silicon-rich silicon oxide Opt Exp 2009 17 1906 10.1364/oe.17.000906 19158905

[B12] Cueff S Labbé C Cardin J Doualan JL Khomenkova L Hijazi K Jambois O Garrido B Rizk R Efficient energy transfer from Si-nanoclusters to Er ions in silica induced by substrate heating during deposition J Appl Phys 2010 108 064302 10.1063/1.3481375

[B13] Pai PG Chao SS Takagi Y Lucovsky G Infrared spectroscopic study of SiO_x _films produced by plasma enhanced chemical vapor deposition J Vac Sci Technol 1986 A4 689

[B14] Savchyn O Ruhge FR Kik PG Todi RM Coffey KR Nukala H Heinrich H Luminescence-center-mediated excitation as the dominant Er sensitization mechanism in Er-doped silicon-rich SiO2 films Phys Rev B 2007 76 195419

[B15] Hijazi K Rizk R Cardin J Khomenkova L Gourbilleau F Towards an optimum coupling between Er ions and Si-based sensitizers for integrated active photonics J Appl Phys 2009 106 024311 10.1063/1.3177243

[B16] Stoney GG The tension of metallic films deposited by electrolysis Proc R Soc London Ser A 1909 82 172 10.1098/rspa.1909.0021

[B17] La Magna A Nicotra G Bongiorno C Spinella C Grimaldi MG Rimini E Caristia L Coffa S Role of the internal strain on the incomplete Si/SiO_2 _phase separation in substoichiometric silicon oxide films Appl Phys Lett 2007 90 183101 10.1063/1.2734398

[B18] Massoud HZ Przewlocki HM Effects of stress annealing in nitrogen on the index of refraction of silicon dioxide layers in metal-oxide-semiconductor devices J Appl Phys 2002 92 2202 10.1063/1.1489500

[B19] Cueff S Labbé C Dierre B Cardin J Khomenkova L Fabbri F Sekiguchi T Rizk R Cathodoluminescence and photoluminescence comparative study of Er-doped Si-rich silicon oxide J Nanophoton 2011 5 051504 10.1117/1.3549701

[B20] Cueff S Labbé Cardin J Rizk R Impact of the annealing temperature on the optical performances of Er-doped Si-rich Silica systems IOP Conf Ser: Mater Sci Eng 2009 6 012021

[B21] Cueff S Labbé C Dierre B Fabbri F Sekiguchi T Portier X Rizk R Investigation of emitting centers in SiO_2 _co-doped with silicon nanoclusters and Er^3+ ^ions by cathodoluminescence technique J Appl Phys 2010 108 113504 10.1063/1.3517091

[B22] Franzò G Pecora E Priolo F Iacona F Role of the Si excess on the excitation of Er doped SiO_x_ Appl Phys Lett 2007 90 183102 10.1063/1.2734505

